# Tracking the best reference genes for RT-qPCR data normalization in filamentous fungi

**DOI:** 10.1186/s12864-015-1224-y

**Published:** 2015-02-14

**Authors:** Agustina Llanos, Jean Marie François, Jean-Luc Parrou

**Affiliations:** Université de Toulouse; INSA, UPS, INP; LISBP, 135 Avenue de Rangueil, F-31077 Toulouse, France; INRA, UMR792 Ingénierie des Systèmes Biologiques et des Procédés, F-31400 Toulouse, France; CNRS, UMR5504, F-31400 Toulouse, France; Cinabio-Adisseo France S.A.S., 135 Avenue de Rangueil, 31077 Toulouse, France

**Keywords:** Filamentous fungi, Talaromyces, RNA-seq, RT-qPCR, Reference genes, Normalization, Gene expression

## Abstract

**Background:**

A critical step in the RT-qPCR workflow for studying gene expression is data normalization, one of the strategies being the use of reference genes. This study aimed to identify and validate a selection of reference genes for relative quantification in *Talaromyces versatilis*, a relevant industrial filamentous fungus. Beyond *T. versatilis*, this study also aimed to propose reference genes that are applicable more widely for RT-qPCR data normalization in filamentous fungi.

**Results:**

A selection of stable, potential reference genes was carried out *in silico* from RNA-seq based transcriptomic data obtained from *T. versatilis*. A dozen functionally unrelated candidate genes were analysed by RT-qPCR assays over more than 30 relevant culture conditions. By using geNorm, we showed that most of these candidate genes had stable transcript levels in most of the conditions, from growth environments to conidial germination. The overall robustness of these genes was explored further by showing that any combination of 3 of them led to minimal normalization bias. To extend the relevance of the study beyond *T. versatilis*, we challenged their stability together with sixteen other classically used genes such as β-tubulin or actin, in a representative sample of about 100 RNA-seq datasets. These datasets were obtained from 18 phylogenetically distant filamentous fungi exposed to prevalent experimental conditions. Although this wide analysis demonstrated that each of the chosen genes exhibited sporadic up- or down-regulation, their hierarchical clustering allowed the identification of a promising group of 6 genes, which presented weak expression changes and no tendency to up- or down-regulation over the whole set of conditions. This group included *ubcB*, *sac7, fis1* and *sarA* genes, as well as *TFC1* and *UBC6* that were previously validated for their use in *S. cerevisiae*.

**Conclusions:**

We propose a set of 6 genes that can be used as reference genes in RT-qPCR data normalization in any field of fungal biology. However, we recommend that the uniform transcription of these genes is tested by systematic experimental validation and to use the geometric averaging of at least 3 of the best ones. This will minimize the bias in normalization and will support trustworthy biological conclusions.

**Electronic supplementary material:**

The online version of this article (doi:10.1186/s12864-015-1224-y) contains supplementary material, which is available to authorized users.

## Background

Filamentous fungi are involved in several natural and industrial processes. They have long been used for the production of additives used in food and beverages [1]. Some fungi produce enzymes that degrade lignocellulosic material with applications in food, feed, textile, pulp and paper industries [2,3]. The genera *Penicillium* and *Aspergillus* are the most biotechnologically important fungi, due to their ability to produce secondary metabolites, organic acids or enzymes, but recent genome sequences of hundreds of fungal species indicate that the potential of fungi has been substantially underestimated [4,5]. *Talaromyces* is another industrially relevant genus closely related to *Penicillium* [6], among which *Talaromyces versatilis* is exploited for the production of a commercial cocktail called “Rovabio Excel™” that is used as feed additive for enhancing digestibility of cereal based diets. However, fungi are not restricted to biotechnologically relevant organisms. Recent estimates suggest that more than 5 million fungal species exist in this monophyletic kingdom, the huge majority being in the Ascomycota and Basidiomycota phyla [7]. Fungi have considerable impact in agriculture, as fungi are capable of intimate symbiotic associations with plants as in the case of *Rhizophagus irregularis* [8] while some species are economically serious plant pathogens [9], *e.g. Leptosphaeria maculans* [10], *Blumeria graminis* [11], *Rhizoctonia solani* [9,12], *Magnaporthe grisea* [13]. Finally, they not only draw interest as pathogens of invertebrate animals, but they are also harmful for human health, as for example with the production of mycotoxins and allergens [14], with several species, including *Aspergillus fumigatus*, causing invasive disease [15]. For all these reasons, and aided by extraordinary advances in genome sequencing facilities [16,17], there has been a tremendous effort to pursue the sequencing of filamentous fungi. The availability of genomic sequences from several fungi has favoured the rapid development of high throughput transcriptomic studies and functional genomics analysis.

Better knowledge of gene function usually begins by investigating expression of the genes of interest (GOIs) under a broad set of culture conditions. Several techniques have been developed to measure expression levels, among which the coupling between reverse transcription and quantitative (real-time) PCR (RT-qPCR) appears to be the most appropriate to study limited numbers of genes in large sets of conditions [18,19]. Significant technical advances made this mRNA quantification method very accessible, highly specific and sensitive, but numerous critical issues remain that limit the ability to draw meaningful conclusions [20]. The Minimum Information for Publication of Quantitative Real-Time PCR Experiments (MIQE) guidelines help in the design of experiments, to keep track of the experimental data and to improve analysis [21,22]. Importantly, and no matter what the technique for measuring gene expression is, data normalization is a critical step. Performance and pitfalls of the different normalization strategies has already been compared in a number of dedicated review articles [19,23]. Few articles promote the use of external controls [24,25], a normalization strategy stimulated by the ERCC or EQUAL-quant programs [26,27], and especially relevant for the assessment of technical robustness in clinical and biological diagnostic laboratories. But normalization of gene expression levels by reference genes (internal controls) is most certainly the gold standard, even if it is now clearly established that the use of a single gene is not acceptable, as there is not a single gene that has a stable transcript level over all kinds of culture conditions or among different cell types [28-31]. The main challenge concerning these internal controls is the circular problem in evaluating expression stability of a candidate normalization gene [32], *i.e*. how can the expression stability of a candidate be evaluated if no reliable measure is available to normalize the candidate? To overcome this circular problem, Vandesompele et al. [33] first developed more than ten years ago a method called geNorm, which allows the evaluation and ranking of candidate reference genes in terms of expression stability (or suitability as normalizing gene). In a subsequent step, the algorithm is able to indicate how many reference genes are optimally required to remove most of the technical variation. Other algorithms were then developed (e.g. Normfinder [32] or BestKeeper [34]) and were presented in a comprehensive survey [28]. Good practice in data normalization for gene expression analysis therefore relies on the identification, experimental validation and use of several reference genes. In filamentous fungi, such efforts have been observed with recent publications dedicated to the validation of suitable reference genes under specific experimental contexts [35-45]. In Zhou’s work [39], *cypB* and *crzA* were evaluated because of their stability in transcriptomic datasets. Similarly, Kim and Yun [46] selected 8 reference genes from transcriptomic data available with *Fusarium graminearum*. Such an approach was an exception, as most often, authors have evaluated more classic “housekeeping genes” encoding for example actin, glyceraldehyde-3-phosphate dehydrogenase or β-tubulin, which are still and too frequently used as single, non-validated reference genes.

During the course of the RNA-seq based transcriptomic analysis of the industrial strain *T. versatilis* exposed to wheat straw, it was found that most of the classical reference genes exhibited expression changes in the presence of this lignocellulosic substrate (unpublished data). This finding prompted the formulation of a list of putative reference genes and validation of their expression stability in *T. versatilis* cultivated under more than 30 different relevant conditions, following the MIQE guidelines for robust and reliable RT-qPCR expression data acquisition and treatment. Finally, 90 RNA-seq based transcriptomic datasets from 18 phylogenetically distant filamentous fungi were scrutinized, including datasets from industrially important or model species as well as plant or animal interacting fungi, to demonstrate that some of the candidate genes suitable for *T. versatilis* can be proposed as promising reference genes for data normalization in RT-qPCR analysis in other filamentous fungi.

## Methods

### Strain and culture conditions

The industrial strain used in this work, *Talaromyces versatilis* (basionyme *Penicillium funiculosum*, IMI378536), is an ADISSEO proprietary strain (patent no. W0 99/57325). Spores of *T. versatilis* were obtained by growing the strain on Potato Dextrose Agar (PDA) plates and the spores were used to inoculate liquid medium. The minimal medium (MM) contained for 1 L: 1.9 g KH_2_P0_4_, 0.65 g KCl, 0.65 g MgSO_4_, 12.5 mg ZnSO_4_, 12.5 mg MnCl_2_, 12.5 mg FeSO_4_, 5 g NH_4_Cl. The MM was supplemented with 10 g/L glucose as the sole carbon source, unless otherwise stated. The pH was adjusted to 6.0 with 50 mM KH_2_P0_4_. The liquid medium was inoculated with 2 × 10^5^ spores/mL in Erlenmeyer flasks. The cultures were carried out at 30°C and agitated at 150 rpm for 48 h.

### Mycelia samples

A summary table of the culture conditions is presented as Additional file 1. To prepare mycelia samples of *T. versatilis* exposed to different carbon sources, the mycelia were grown for 48 h in MM broth culture and were filtered through Miracloth (Merck), washed with MM without carbon source and transferred to fresh media containing the desired carbon sources. The cultures were incubated from 30 minutes to 2 hours for growth on monosaccharides (arabinose 0.2% (w/v) or xylose 0.2%) or disaccharides (cellobiose 0.2%, xylobiose 0.2% or thio-gentiobiose 0.2%), or up to 24 hours for the cultures containing complex carbon sources (Avicel 1%, Arbocel 1%, beechwood xylan 1%, ball-milled wheat straw 1% or micronized wheat bran 1%). For exposure to stress, the *T. versatilis* mycelia grown for 48 h in MM medium were filtered through Miracloth, washed with MM without carbon source and transferred to MM supplemented with 0.5 M KCl for salt stress, MM without glucose for carbon starvation, MM without ammonium for nitrogen starvation, and MM with the pH adjusted to 2 or 8 for pH stress. The cultures were incubated at 30°C for 1 h before sampling. For the temperature stress, the mycelia were similarly collected and transferred to a pre-heated MM broth culture for an additional 1 h at 40°C. Samples of about 50 mg of mycelium were then collected by filtration through Miracloth and flash frozen in liquid nitrogen.

The conidia at different developmental stages were prepared after inoculating MM with 2 × 10^5^ spores/mL. Samples were harvested by centrifugation at 3000 g for 2 min, after incubation of the spores at 30°C, 150 rpm for 2 h (no morphological change), 4 h (early swelling), 8 h (late swelling), 12 h (germ tube on one side of the conidia) and 16 h (hyphae already visible). 500 μL of pre-heated RNA extraction buffer (NaCl 0.6 M, sodium acetate 0.2 M, EDTA 0.1 M, SDS 4%) were added to each sample before immersing in liquid nitrogen.

### RNA extraction and cDNA synthesis

Mycelia and conidia samples were mechanically disrupted using the TissueLyser II (Qiagen). Frozen mycelia samples were disrupted with a single 5 mm stainless steel bead (Qiagen), whereas thawed conidia preparations were mixed with approx. 150 μL of 625 μm glass beads (Sigma). Both were submitted to two high-speed shaking cycles of 3 minutes at 30 Hz. Total RNA was isolated from disrupted mycelia samples using the GeneJET Plant RNA Purification Mini Kit (Thermo). An on-column DNase I treatment (Thermo – Reference #EN0521) was added to the protocol, applying 100 μL of the DNase I mix (50 μL of DNase I, 10 μL of 10 X buffer and 40 μL of nuclease-free water) to the column after the first wash, for a 30 minutes incubation at room temperature and final wash with the wash buffer I. The remaining of the protocol was performed as recommended by the Supplier. For conidia and germinating conidia samples, total RNA was isolated after transfer of the liquid, beads-free phase to a tube containing 1 mL of TRIzol reagent (Invitrogen). 0.25 mL of chloroform was added to each sample and the tubes were incubated for 5 minutes at room temperature and then vortexed. The tubes were centrifuged at 16000 g for 15 minutes. The aqueous phase (approx. 750 μL) was transferred to a clean tube and 1 volume of isopropanol was added. The samples were mixed by inverting the tubes several times. The tubes were incubated at room temperature for 10 minutes and centrifuged at 16000 g for 10 minutes. The supernatant was removed and the pellet was washed with 1 mL of 70% (v/v) ethanol and centrifuged once again at 16000 g for 10 minutes. The ethanol was discarded and the pellet was left to dry. Each pellet was resuspended in 50 μL of nuclease-free water. A clean-up protocol using the RNeasy Mini Kit (Qiagen) and on-column DNase I treatment (Thermo) was then performed on these RNA samples.

The quantification of the RNA samples was assessed by using the ND-1000 UV-visible light spectrophotometer (NanoDrop Technologies) while the Bioanalyzer 2100 with the RNA 6000 Nano LabChip kit (Agilent) was used to certify RNA integrity. Only RNA samples with 260/280 nm wavelength ratio of approximately 2 and 260/230 nm wavelength ratio greater than 2 were retained for analysis. Synthesis of cDNA was performed using the PrimeScript First Strand cDNA Synthesis Kit (Takara), following the Manufacturers’ protocol. One microgram of total RNA from mycelia samples and 100 ng of total RNA from conidia and germinating conidia were used for the cDNA synthesis reaction. The cDNA was diluted 1:10 with water and stored at −20°C.

### Primer design and validation

Primers were designed using Vector NTI advance v11 (Life Technologies) with melting temperature of 58─60°C, length of 18─25 bp and GC content of 50─60%. All except R7 and R9 (see Table 1) possess one to several introns in their sequence, which allowed designing the primers at the exon-exon junctions to minimise the amplification of contaminant gDNA. Amplicon sizes ranged between 70 and 200 bp. Reaction efficiency for each pair of primers was tested by the dilution series method using a mix of cDNA samples as the template. The efficiency of validated primer pairs focused around 100% (Table 1).

**Table 1:** **List of putative reference genes and genes of interest**

**Name**	**Annotation**	**GO terms**	**Pathway**	**Primer sequence**	**Primers efficiency**	**Amplicon size**
R1	DUF221 domain protein (*DUF221*)	Vacuolar membrane (GO:0005774)	Transmembrane protein with unknown function	Fw: CGGAACGCCCCATTGACC	95.1%	126 bp
				Rv: TTGGATGCTTATGTTTTGCTCTCG		
R2	Ubiquitin carrier protein (*ubcB*)	Ligase activity (GO:0016874)	Involved in the ubiquitin mediated proteolysis	Fw: TCGTTGAGTAGACTCTGAATGCTG	99.2%	125 bp
		Cellular response to stress (GO:0033554)				
				Rv: AGCCAGATGTTCCACCCG		
		Cytoplasm (GO:0005737)				
R3	CECR1 family adenosine deaminase (*ADA*)	Adenosine deaminase activity (GO:0004000)	Involved in the purin metabolism	Fw: CTGCGCAATGCAAAGTCATGTCTCTG	100.7%	97 bp
				Rv: CCCAGGTCGAAGATCCCTTTATCCA		
R4	Mitochondrial membrane fission protein (*fis1*)	Metal ion binding (GO:0046872)	Mitochondrial complex that promotes mitochondrial fission	Fw: GTTCAACTACGCCTGGGGACTC	101.1%	91 bp
		Mitochondrial fission (GO:0000266)				
				Rv: AGCGGTGCGAAAAATCTGGG		
		Membrane (GO:0016020)				
R5	Copper-transporting ATPase (*Cu-ATPase*)	Nucleotide binding (GO:0000166)		Fw: TGGTGCCCTGTGCCAACTCTCCCAGTC	103.6%	78 bp
		Cellular metal ion homeostasis (GO:0006875)				
				Rv: TTGCTGCGGGTGCTTTTG		
		Membrane (GO:0016020)				
R6	Cohesin complex subunit (*psm1*)	DNA secondary structure binding (GO:0000217)	Involved in chromosomes segregation during mitosis	Fw: GTATTTGCGGAGATCCAGAGTGAG	93.1%	102 bp
		Mitotic sister chromatid segregation (GO:0000070)		Rv: TTGAAGACGGGTCTGTTCCA		
		Nucleus (GO:0005634)				
R7	Spo7-like protein (*spo7*)	Phosphatase activity (GO:0016791)	Involved in the spore formation process	Fw: GCCGATGGTGCTGATGTTGG	102.5%	110 bp
		Sporulation (GO:0043934)				
				Rv: AGAACGCCAACGAGCCCG		
		Integral to membrane (GO:0016021)				
R8	SAGA-like transcriptional regulatory complex subunit Spt3 (*spt3*)	Transferase activity (GO:0016740)	Component of the nuclosomal histone acetyltransferase (SAGA) complex	Fw: ACGACTTGTTGGCGGACG	96.3%	95 bp
		Chromatin modification (GO:0016568)				
				Rv: GAGATTCAGCAGATGATGTTTGTC		
		Nucleus (GO:0005634)				
R9	DUF500 domain protein (*DUF500*)	Actin filament organization (GO:0007015)	Cytoskeleton organization	Fw: ACTTGGCCGGTTGTGCGTTC	98.5%	101 bp
		Cytoplasm (GO:0005737)		Rv: TTGGTGTTCCGGCGGCTG		
R10	Rho GTPase activator (*sac7*)	Rho GTPase activator activity (GO:0005100)	Involved in signal transduction	Fw: AGGAGGATGAAAGTAAAGGACCCC	100.5%	159 bp
		Small GTPase mediated signal transduction (GO:0007264)				
				Rv: AAACCCCACACTTGGCGAC		
		Intracellular (GO:0005622)				
R11	AP-2 adaptor complex subunit beta (*AP-2 β*)	Transporter activity (GO:0005215)	Involved in chlatrin-dependent endocytosis	Fw: TTTCGCACATAGGGGTCG	98.4%	148 bp
				Rv: TTTTGGTCGATGATATGGACG		
R12	Protein translocation complex componenet (*npl1*)	Protein transporter activity (GO:0008565)	Involved in the protein progression in endoplasmic reticulum	Fw: CGCTGGAACAAGAAAAATACG	98.2%	117 bp
		Post-translational protein targeting to membrane (GO:0006620)				
				Rv: ACGAACGATATGCGCCAA		
		Endoplasmic reticulum (GO:0005783)				
*β-tub*	Beta-tubulin	Nucleotide binding (GO:0000166)	Cytoskeleton	Fw: GTTCTGGACGTTGCGCATCTG	97.2%	110 bp
		Cytoskeleton (GO:0005856)				
				Rv: TGATGGCCGCTTCTGACTTCC		
abf-B2*	Arabinofuranosidase-B2	Hydrolase activity, acting on glycosyl bonds (GO:0016798)	Sugar metabolism	Fw: CGGAGCTTGGGTGAGATGGTTC	103.6%	112 bp
		Carbohydrate metabolic process (GO:0005975)		Rv: CGGCGGCGTTGCTAATGC		
		Extracellular region (GO:0005576)				
xynC*	Xylanase C	Hydrolase activity, acting on glycosyl bonds (GO:0016798)	Sugar metabolism	Fw: CAAATGGCGACAATGGCG	94.4%	104 bp
		Xylan metabolic process (GO:0045493)		Rv: TGAGTACGTGACAGTCTGTGCATTG		
		Extracellular region (GO:0005576)				

The annotation and GO terms were taken from the *Talaromyces versatilis* genome (basionyme *Penicillium funiculosum*, ADISSEO proprietary sequence, unpublished). Forward (Fw) and reverse (Rv) primer sequences used for RT-qPCR. The two genes at the bottom of the table marked with an asterisk (*) correspond to the genes of interest.

### qPCR

The qPCR was performed in a CFX96 Real Time PCR Detection System (Bio-Rad), using 96-well white PCR plate (Thermo) sealed with ABsolute qPCR seals (Thermo). The reaction mix consisted of 7.5 μL of the DyNamo ColorFlash SYBR Green master mix (Thermo), 300 nM of each primer and 3 μL of the 1:10 diluted cDNA in a final volume of 15 μL. The PCR reaction cycle was: initial denaturation for 7 min at 95°C, followed by 40 cycles of 10 seconds at 95°C and 30 seconds at 60°C. A melting curve was performed at the end of the qPCR run, increasing the temperature in a stepwise fashion by 0.5°C every 5 seconds, from 65°C to 95°C. Each RT-qPCR reaction was performed in technical triplicate. Two control samples were included for each primer pair tested; the no template control (NTC) and *T. versatilis* genomic DNA. For each sample, a ValidPrime Assay (VPA), consisting of a pair of primers that bind to a non-transcribed intergenic region identified from RNA-seq data, was also included to detect and quantify the presence of contaminating gDNA [47]. The primers for the VPA were; 5′ACCGAATGGCACCGAGTTGG 3′ and 5′AATGGAGGAAGCGTGCCGTG 3′. As gDNA contamination rarely exceeded 1%, the RT-qPCR data were directly analysed using the CFX Manager software (Bio-Rad).

### Stability analysis

The stability of putative reference genes was assessed using the geNorm VBA applet for Microsoft Excel [33]. geNorm allows the calculation for each reference gene of the gene expression stability value M, which is the average pairwise variation of a particular gene with all other control genes, the most stable genes presenting the lowest M values. To determine the optimal number of genes that are required for an accurate normalization, the normalization factors (NF_n,_ based on the geometric mean of the *n* most stable genes) were calculated by stepwise inclusion of the most stably expressed genes. Pairwise variations (V_(n/n+1)_) between NF_n_ and NF_n+1_ were then calculated to determine the effect of adding the (*n + 1*)^th^ gene. If the V_n/n+1_ is superior to the cut-off value 0.15, the addition of the (*n + 1*)^th^ gene has a significant effect on normalization quality and should preferably be included for calculation of a reliable normalization factor.

### *In*-*silico* analysis of RNA-seq data

Three RNA-seq datasets from the industrial *T. versatilis* were at our disposal (unpublished data) and were prepared from: 1) growth of the mycelium on MM for 48 h (reference condition); 2) transfer of the water-rinsed mycelium to MM with ball-milled wheat straw 1% (w/v) as carbon source and sampling after 24 h; 3) direct addition of glucose at 1% final concentration to the mycelium exposed to wheat straw, and sampling after 5 h. These RNA-seq data were used for the pre-selection of stable genes (fold change (FC) equal to one, see Additional file 2) after calculating the FC as follow: RPKM (Reads Per Kilobase of exon model per Million mapped reads) value in the sample of interest / RPKM in the reference condition, for each gene. Similarly, FC for candidate reference genes were calculated from RNA-seq data publicly available at the NCBI GEO database [48,49]. To identify the homologues of *T. versatilis* selected reference genes in the different fungi, a standard protein BLAST (blastp) using the amino-acid sequence from *T. versatilis* was performed against protein databases, specifying the organism. Each homologous sequence was then used for a reciprocal BLAST against the *T. versatilis* database in order to confirm the accuracy of the result. The detailed list of locus tags for each gene in every fungus is available in the Additional file 3. For each GOI in these studies, the ratio between the expression in a condition of interest and the expression in the control condition was calculated. Collected datasets were from *Trichoderma reesei* ([50], accession #GSE44648), *Aspergillus niger* ([51], #GSE33852), *Aspergillus flavus* ([52,53], #GSE40202 and #GSE30031), *Aspergillus fumigatus* (#GSE30579), *Aspergillus oryzae* ([54], #GSE18851), *Aspergillus nidulans* ([55], #GSE44100), *Blumeria graminis* ([11], #GSE43163), *Colletotrichum graminicola* ([56], # GSE34632), *Colletotrichum higginsianum* ([56], #GSE33683), *Fibroporia radiculosa* ([57], #GSE35333), *Magnaporthe oryzae* ([58], #GSE30327), *Neurospora crassa* ([55], #GSE44100), ([59], #GSE35227), ([60], #GSE36719), *Pyronema omphalodes* ([61], #GSE41631), *Rhizoctonia solani* ([12], #GSE32577), *Sordaria macrospora* ([62], #GSE33668). We also accessed unpublished data from *Rhizophagus irregularis* ([7], #SRX375378 at NCBI Short Read Archive) and *Leptosphaeria maculans* (personal communication from T. Rouxel, INRA-Bioger, Thiverval-Grignon, France).

### Gene expression and statistical analyses

Three independent cultures of *T. versatilis* were carried out to perform RNA-seq. For reference gene stability analysis by RT-qPCR, cultures of *T. versatilis* in the different conditions were performed in duplicate. qPCR assays were performed in technical triplicates. Inter-run calibrators were included in each qPCR plate. The RT-qPCR data were directly analysed using the CFX Manager software (Bio-Rad), which allows inter-run calibrations, efficiency correction, normalization with multiple reference genes and calculation of ratios with (technical) errors propagation. As advised for final calculation of FC values from biological replicates [20], statistics (mean ± SD) were assessed from FC values obtained from biological replicates. Other statistical analyses were conducted by using the STATGRAPHICS Centurion 16 software. This included: the ANOVA on relative FC values (Three-level, nested ANOVA with ‘genes’ as the first level, ‘culture conditions’ as the second level and ‘biological replicates’ as the third level); the Hierarchical Ascendant Classification (HAC) that was performed according to the Ward method, using default parameters (standardization of the data and squared Euclidean distances); the graphical representation of box plots.

## Results and discussion

### Selection of candidate reference genes from *T. versatilis* RNA-seq datasets

In a preliminary study on the industrial *Talaromyces versatilis* strain IMI378536, RNA-seq data were generated to analyse the transcriptome of this filamentous fungus on wheat straw (unpublished data, property of ADISSEO SAS). An *in silico* screen for genes that showed no differential expression between glucose and wheat straw, was used to select about a hundred genes with fold-changes close to one, indicating stability of transcript levels under those conditions. From this pre-selection, genes were discarded because of anti-sense transcription, alternative splicing events, as well as very low expression level (RPKM below 15). The design of primers and their experimental validation by RT-qPCR were then performed on a residual list of 20 candidate genes taking care to avoid their participation in similar cellular functions to minimise the risk of co-regulation under culture conditions of interest. Finally, 12 putative reference genes were selected whose primers led to good reaction efficiency. As shown in Table 1, this selection included genes involved in intracellular signalling, vesicular trafficking, metal transport, cytoskeleton organization or protein ubiquitinylation, but quite surprisingly, it did not contain any gene implicated in the central carbon metabolism. To this list, the gene encoding β-tubulin was also included, as it is frequently used for RT-qPCR data normalization [63-67].

### Evaluation of the stability of candidate genes expression in *T. versatilis* cultivated under a large set of conditions

**Figure 1:**
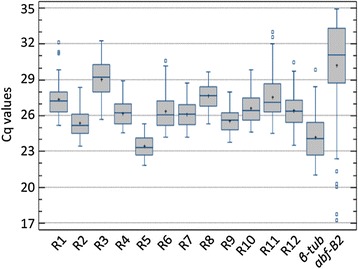
**Distribution of the raw Cq values.** For each gene, the box-plot gathered all the 66 raw Cq values obtained from the 33 duplicated culture conditions of *T. versatilis*. The lower and upper boundaries of the box (interquartile) represent the 25^th^ and the 75^th^ percentile, respectively. The line within the box corresponds to the median and the cross to the mean of the distribution, while the whiskers indicate the highest and the lowest Cq values, with the exception of the outliers that are represented by the squares outside the whiskers.

To evaluate whether the 13 candidate genes harboured a stable transcript level and could be used as proper internal control for data normalization in RT-qPCR gene expression analysis, their transcript levels were quantified by this technique in more than 30 different conditions (Additional file 1). Growth was explored in the presence of different carbon sources (from monosaccharides to complex plant cell wall polymers), temperature, pH and salt stresses, as well as to carbon and nitrogen starvation. In addition, transcript levels of these genes were monitored during conidial germination, as this developmental process is a particularly interesting aspect of fungal biology [68-70]. The raw Cq values of the 13 genes were therefore collected under 33 conditions and compiled in the box plot (Figure 1). Most of these genes showed a compact distribution of Cq values, with less than 2 Cq between the 1^st^ and 3^rd^ quartiles, indicating relatively low variation of the transcript level among the different conditions (*i.e*. less than 4-fold differential expression for the middle fifty). Some of them, R3 (*ADA*), R11 (*AP*-*2β*) as well as the *β*-*tub* gene displayed slightly higher dispersion of their Cq values. The genes R3 and *β*-*tub* also exhibited weaker and stronger transcript levels, respectively, with *approx*. 100-fold differential average transcript level between each other. Besides these 2 candidates, quite similar average expression levels for the remaining 11 genes, with raw Cq values around 25, were observed. This average expression level was acceptable for robust RT-qPCR assays and normalization, based on the validated reaction efficiencies and the possibility to amplify target cDNA over several logs of concentration. However, this was contrary to the notion that the transcript level of the ideal reference gene must be close to the average transcript level of the GOIs. That situation cannot occur when the GOIs present very different average transcript levels, or when a single GOI presents either potent repression or strong induction in the same study. As an example, the expression of *abf*-*B2* encoding a GH54 α-L-arabinofuranosidase [71], showed more than 25-fold relative change between the 1^st^ and 3^rd^ quartiles. The huge whiskers of *abf*-*B2*’*s* box (Figure 1) reflected more than four log differential expression, and at least a 5-log differential expression of *abf-B2* between the two extreme conditions was observed.

**Figure 2:**
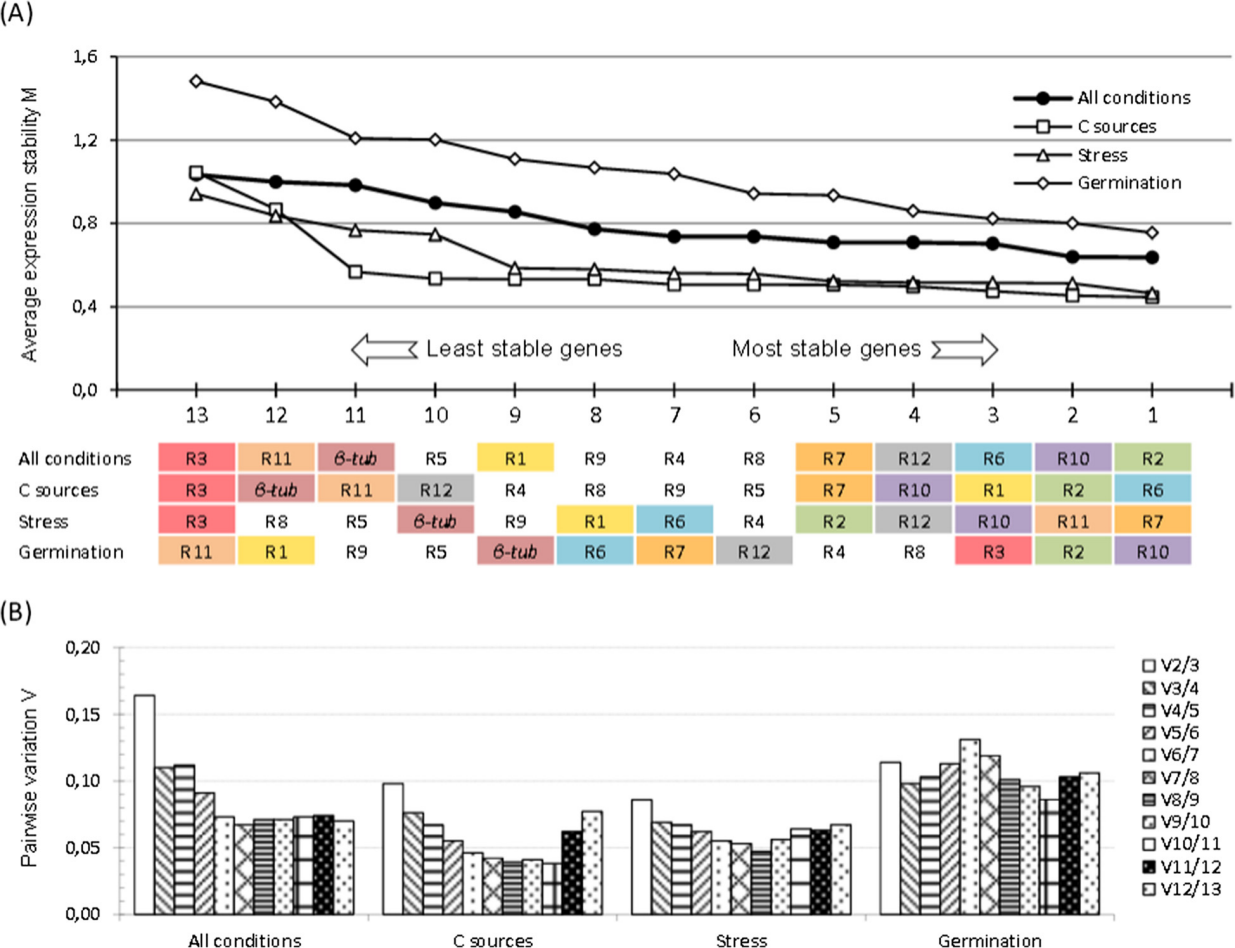
**geNorm**–**based ranking of the putative reference genes. (A)** Genes were ranked from the least stable (on the left) to the most stable (on the right) according to their *M* value (Y axis). This classification was independently performed by using different sets of conditions: the ‘All conditions’ included the whole set of culture conditions studied by RT-qPCR in this work; the ‘C sources’ subset gathered the 18 samples obtained from growth on different sugars; the ‘Stress’ subset corresponded to 6 samples harvested during stress exposition; the ‘Germination’ subset included the 6 germination time points. For each set of conditions, the result of the classification was given below the X-axis (arbitrary colours attributed to each gene for the sake of clarity). **(B)** Result of the pairwise variation analysis between NF_n_ and NF_n+1_ to determine the optimal number of genes for reliable normalization. Values below the 0.15 threshold mean that n genes might be sufficient.

The raw Cq values were transformed to quantities with efficiency correction and then analysed with the geNorm algorithm to rank the 13 candidate genes according to their *M* value and to ascertain their expression stability over a specified set of conditions (Figure 2A). When considering the whole set of conditions that were investigated (“all conditions”), the *M* value of these 13 genes (numbered 1 to 13 on the X axis in Figure 2A) was below the recommended threshold of 1.5, even for the *β-tub* gene, but also R3 and R11, which ranked amongst the least stable genes in agreement with the behaviour that was reported in Figure 1. To reinforce this result, RefFinder (http://www.leonxie.com/referencegene.php), a web-based tool that integrates the currently available major computational programs (geNorm [33], Normfinder [32], BestKeeper [34] and the comparative ΔCt method [72]) was also used. As can be seen in the Additional file 4, these algorithms led to similar classifications with the notable exception of Bestkeeper that ranked R5 and *β-tub* as the most stable genes. Also surprisingly, geNorm from RefFinder did not lead to the same output as we got from our geNorm interface, which certainly relied on different versions of the algorithm. Similar ranking could nevertheless be observed with respect to the most stable candidates (R2, R10, R6, R12 (Figure 2A) vs. R10, R12, R2, R4 (geNorm from RefFinder)) or the least stable ones (R3, R11, *β-tub*, R5 (Figure 2A) vs. *β-tub*, R3, R11, R5 (RefFinder)). The fact that these algorithms, in particular geNorm versus NormFinder and ΔCt method, did not yield to identical results was not so surprising. As will be discussed in the next section with the classification of the genes in subsets of conditions, we indeed observed an unexpectedly uniform stability of the candidate reference genes, suggesting that they are all almost as good as each other. This is most likely the reason why these different algorithms could not propose clear-cut, identical classifications of these genes.

Still using geNorm, the optimal number of reference genes required for accurate normalization in the “all conditions” set (Figure 2B) was evaluated. Vandesompele and coworkers [33] recommended a cut-off value at 0.15 for the pairwise variation value (V_n/(n+1)_), below which the inclusion of an additional gene does not result in a significant improvement of the normalization. According to this criterion, the V_2/3_ and V_3/4_ values indicated that three genes, *i.e*. R2 (*ubcB*), R10 (*sac7*) and R6 (*psm1*), were sufficient for accurate normalization of transcript levels in any of the samples examined.

### Evaluating gene expression stability in subsets of conditions

The stability of transcript levels was similarly analysed in subsets of selected conditions, *i.e*. samples from mycelium grown in different carbon sources (‘C sources’ subset), samples from mycelium exposed to different stress conditions (‘Stress’ subset) and samples harvested during conidial germination (‘Germination’ subset) (Figure 2A). While R1, R11 and R3 were amongst the least stable genes when analysing the whole set of conditions, they classified amongst the 3 best genes in the ‘C sources’, ‘Stress’ and ‘Germination’ subsets, respectively. This reorganisation of the ranking, when conditions changed, could be explained by the uniformly stable transcript levels from these genes, particularly in the ‘C sources’ and ‘Stress’ subsets, which led to low and stable *M* values for 11 and 9 genes amongst the 13 candidates, respectively. This remarkable stability for most of the genes was also supported by the pairwise variation values (Figure 2B), which indicated that only two genes could ultimately be used for robust normalization in these ‘C-sources’ (R6 and R2), ‘Stress’ (R7 and R11) and ‘Germination’ (R10 and R2) subsets. In the first two subsets, the stepwise inclusion of reference genes led to continuous decrease of the V value, until the inclusion of the least stable genes reversed the tendency. These values were nevertheless always below the 0.15 cut-off value, confirming the extreme stability of transcript levels from these genes.

**Figure 3:**
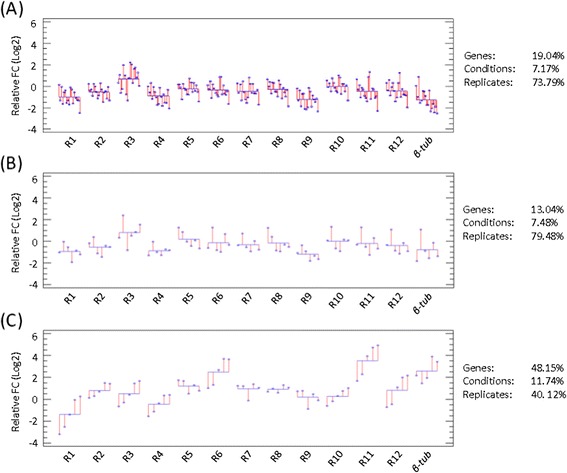
**Hierarchical ANOVA of the putative reference genes.** Three-level, nested ANOVA with ‘genes’ as the first level, ‘culture conditions’ as the second level and ‘biological replicates’ as the third level. As in Figure 2, this ANOVA was carried out using different sets of conditions: ‘C sources’ subset **(A)**; ‘Stress’ subset **(B)**; ‘Germination’ subset **(C)**. Left graph: relative expression values (Log _(base 2)_) as a function of the different conditions for the different genes, taking as the control conditions the glucose sample (A & B subsets) and the T0 time-point for spore germination (C subset). Two values were used for each condition (*i.e.* duplicated experiment). Right panel: partitioning of the variance into the three levels (in %).

In the context of conidial germination, the identification of reliable reference genes was first challenged by the difficulty of producing good quality RNA samples. The influence of RNA quality on reproducibility of measured transcript levels was recently reviewed [73,74], highlighting that the process of normalization does not completely resolve the bias of using compromised RNA quality on the final results. In our hands, only the use of the TriZol reagent secured the mRNA quality standard required for reliable RT-qPCR analysis. This technical prerequisite being fulfilled, the analysis of Cq values in this ‘Germination’ subset showed that the *M* values increased more rapidly than for ‘C-sources’ and ‘Stress’ subsets, indicating higher expression variability of the genes. This was further illustrated by a hierarchical ANOVA of the relative transcript level data (Figure 3), where it was observed that 70 to 80% of the variation for the ‘C-sources’ and ‘Stress’ subsets took place at the level of the biological replicates (Figure 3A & B), supporting the extremely low variation between genes as well as the low influence of conditions on the transcript levels. In contrast, the variation observed between genes strongly increased in the ‘Germination’ subset, to reach about 50% of total variation (Figure 3C), which was particularly emphasized with genes such as R6 and R11 that exhibited a strong bias (higher expression and activation during germination). The genes R10, R2 and R3, which were classified by geNorm as the best reference genes in this specific subset, were used for normalization (see below, NF_(R10, R2, R3)_) and confirmed that R6 and R11 were induced respectively by 6 and 12-fold, 6 hours after the beginning of the germination process (data not shown).

### Robust normalization using the geometric mean of a minimal number of these candidate reference genes

**Figure 4:**
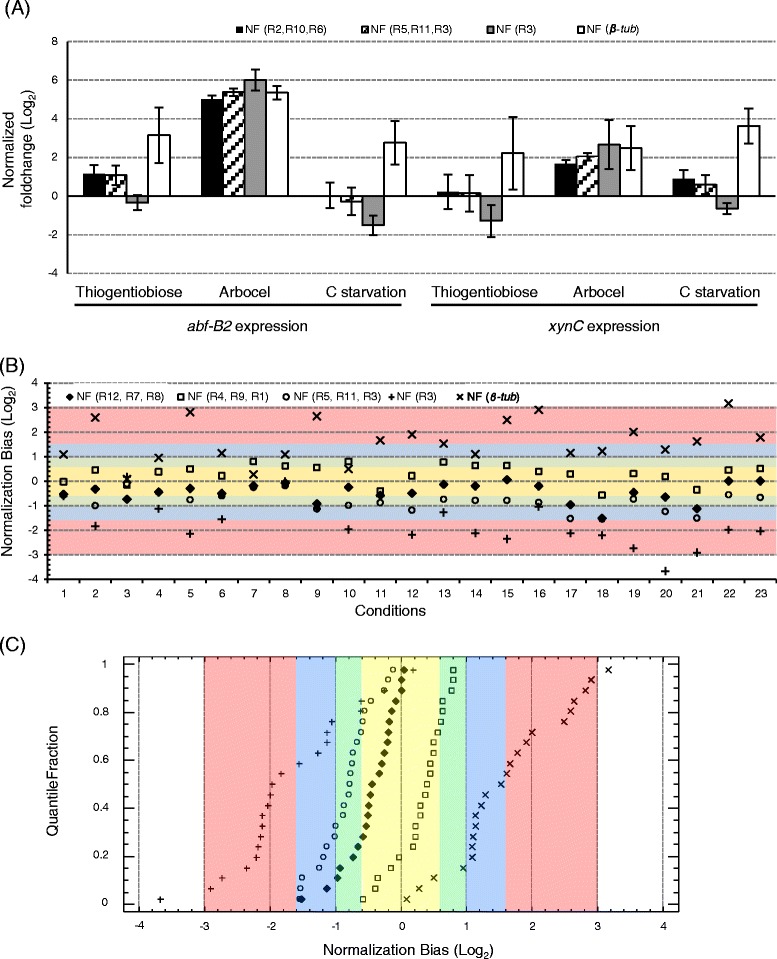
**Normalization bias analysis according to reference genes selection. (A)** Example of normalized transcript levels of *abf-B2* and *xynC* under different culture conditions (Thio-gentiobiose, Arbocel and C starvation samples) using 4 different Normalization Factors (NFs): NF_(R2, R10, R6)_, NF_(R5,R11,R3)_, NF_(R3)_ and NF_(*β-tub*)_. Log _(base 2)_ of FC values on the Y axis (mean ± SD, n = 2 in this experiment) using glucose as the calibrator sample. **(B)** Comparison of NF_(R2, R10, R6)_ to NF calculated from less stable genes, as well as from single genes such as R3 and *β-tub*. For each condition of interest (X axis, see Additional file 1), we calculated a normalization bias (*i.e.* under- or over-estimation of the normalised expression value of GOIs) as the ratio between the theoretically best NF (NF_(R2, R10, R6)_ as determined from geNorm classification by using the entire set of conditions) and NF calculated from other combinations of reference genes. Log _(base 2)_ of the normalization bias is represented on the Y axis. Yellow zone: less than 1.5 fold bias; Green zone: 1.5─2 fold bias; Blue zone: 2─3 fold bias; Red zone: 3─8 fold bias. **(C)** Quantile plot of the normalization bias values for each NF. The normalization bias (Log _(base 2)_) is represented on the X axis and the same colour code used in (B) was applied. The quantile fractions are represented on the Y axis. (♦) NF_(R12,R7,R8)_, (☐) NF_(R4,R9,R1)_, (◯) NF_(R5,R11,R3)_, (+) NF_(R3)_ and (×) NF_(*β-tub*)_.

In order to minimise the risk of bias from using a single gene as a reference, it is preferable to normalize using the geometric mean of multiple reference genes, as it was proposed previously [33]. This normalization bias, *i.e*. under- or over-estimation of the normalised expression value of GOIs, or how the use of different reference genes can impact biological conclusions, was illustrated by studying the transcript level of two relevant genes of *T. versatilis*, namely *abf-B2* encoding a GH54 arabinofuranosidase [71] and *xynC* encoding a GH11 xylanase [75], under three different culture conditions (Figure 4A). Using a theoretically ideal normalization factor (NF_(R2, R10, R6),_ geometric mean of R2, R10 and R6 transcript levels), it was found that the transcript level of *abf-B2* increased by 2.2 and 32-fold upon transfer from glucose to thio-gentiobiose and arbocel, respectively, whereas the transfer to C-starved medium did not cause any significant change. Different regulatory patterns were obtained using single reference genes for normalization. The use of R3, the worst gene according to geNorm classification, led to no-significant change of *abf-B2* transcript level upon transfer to thio-gentiobiose, slightly over-estimated induction on arbocel (64-fold), and indicated that this gene was repressed by 3-fold upon C-starvation. The use of *β-tub* also modified the expression pattern but in contrast to R3, it allowed concluding that *abf-B2* was induced by 9 and 7-fold upon transfer to thio-gentiobiose and C-starvation, respectively. Similar conclusions could be drawn by analysing *xynC*. While transcripts levels of this gene were not significantly affected upon transfer to thio-gentiobiose or C-starved medium when using NF_(R2, R10, R6)_, the use of R3 allowed concluding that this gene is slightly repressed upon transfer to these conditions, while the use of *β-tub* would conclude on clear induction by 5- and 12-fold, respectively. Only in a few circumstances (*e.g*. arbocel sample in this example), the use of single genes for normalization may lead to similar fold change values whatever the reference gene used. To better prove the importance of averaging several, functionally unrelated candidate reference genes to gain a significant reduction of the normalization bias, we gathered together the three worst candidate genes, including R3, to calculate the normalization factor NF_(R5, R11, R3)._ Fold change values reported in Figure 4A clearly showed that the use of NF_(R2, R10, R6)_ and NF_(R5, R11, R3)_ lead to almost identical results. This result discredited the use of single genes such as R3 or *β-tub* (even if their use could, by chance, lead to fairly good conclusions (*e.g*. arbocel condition in this figure)), but it illustrated the strength of geometric averaging multiple genes to smooth individual, sometimes important variations of the transcript level of single reference genes. This is a particularly relevant aspect to avoid incorrect biological interpretation of gene regulation, particularly if the biological significance of subtle differences in fold-changes values is to be considered.

These preliminary, illustrative results prompted us to extend this analysis in our specific set of conditions to obtain a better idea of the frequency and extent of under- or over-estimation of normalized expression value of GOIs by using different reference genes. Also, different sub-optimal combinations of reference genes were challenged by evaluating the normalization bias that might result from their use. We studied here normalization factors (NF) calculated from combinations of three candidates, from less well ranked genes (NF_(R12, R7, R8)_, NF_(R4, R9, R1)_ and NF_(R5, R11, R3)_, respectively). The normalization bias was calculated in each condition as the ratio between a given normalization factor and the theoretically ideal one (NF_(R10, R2, R6)_). The use of the *β-tub* gene alone, which ranked amongst the least stable genes (Figure 2A), altered the quality of the normalization with over-estimation in most of the conditions that were studied (Figure 4B, crosses in the upper blue and red zones). This effect was emphasized in the quantile plot (Figure 4C), as an unbiased response (*i.e*. less than 1.5-fold bias) was observed in less than 10% of the conditions, while 50% of the samples showed more than 3-fold over-estimation. Conversely, the use of R3 alone resulted in 3.0–8.0-fold under-estimation in more than half of the samples tested in our study (Figure 4B & C, lower red zone). We could observe a significant reduction of the normalization bias by averaging R3 in NF_(R5, R11, R3),_ which led to a clear shift of the curve towards the central zone, with more than 70% of the conditions exhibiting less than 2-fold bias while the remaining 6 samples did not exceed 3.0-fold down-estimation (conditions #12, 17, 20 and 21 from the NF_(R5, R11, R3)_ series, blue zone), confirming the results shown on Figure 4A. In most cases, the use of NFs that were calculated from multiple genes led to a minimal normalization bias (yellow-green zone), which was a clear illustration of the prime importance of normalising by the geometric averaging of multiple genes to minimize the bias during normalization of GOIs [33].

### Expression data collection from phylogenetically distant filamentous fungi

To demonstrate the suitability of some of the 12 putative reference genes for RT-qPCR analysis in fungi, RNA-seq based transcriptomic datasets from 18 phylogenetically distant filamentous fungi were interrogated, exploiting web resources such as the GEO portal [48,49]. These datasets covered model fungi [55,59-62], biotechnologically important organisms [50,51,54], agronomically relevant fungi such as symbiotic organisms or plant pathogens ([8,11,12,47-49] and personal communications), and human pathogens [52,53]. This collection corresponded to 90 independent datasets, most of them in triplicates and harvested from a broad variety of experimental conditions, *e.g*. exposure to stress, nutritional source utilisation, fungi-host interactions or development stages (see Additional file 5 for further details). To further strengthen this analysis, genes were included that have been evaluated for their use in *Aspergillus niger* [35] and *Trichoderma reesei* [36]. The 12 genes selected here are henceforth referred to as the ‘R series’ and the additional reference genes were termed ‘C series’ and contained the actin (C1, *act*), aminopeptidase C (C2, *apsC*), cytochrome C oxidase subunit V (C3, *coxV*), glyceraldehyde-3-phosphate dehydrogenase (C4, *gapdh*), glucokinase (C5, *glkA*), glucose-6-phosphate dehydrogenase (C6, *g6pdh*), isocitrate dehydrogenase precursor (C7, *icdA*), phosphofructokinase (C8, *pfkA*), phosphoglucose isomerase (C9, *pgiA*), a secretion associated GTP-binding protein (C10, *sarA*), and the translation elongation factor a1 (C11, *ted1a*). The *β-tub* gene (C12), which was not part of *A. niger* and *T. reesei* studies, was also included in this study as it has been evaluated amongst other putative reference genes in similar studies ([38-43] and Additional file 6). Finally, 4 further genes were added that were homologous to *S. cerevisiae ALG9* (Sc1), *TAF10* (Sc2), *TFC1* (Sc3) and *UBC6* (Sc4), which have been previously validated as good reference genes in this yeast [76]. For every gene, in each specific study, fold changes (FC) were calculated as the ratio between the expression in a condition of interest and the expression in the control condition that was designed in this specific study.

**Figure 5:**
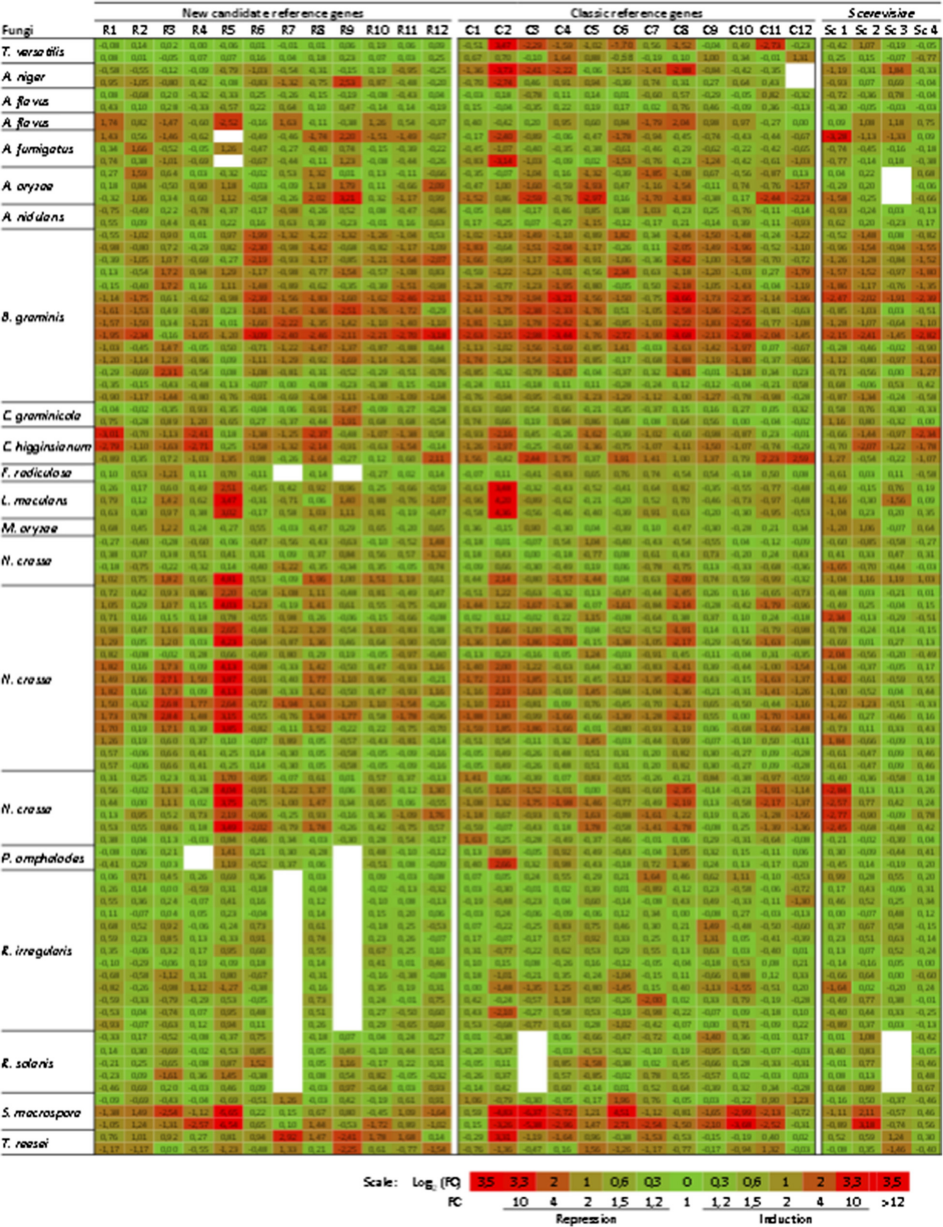
**Heat map of RNA-seq based expression data of putative reference genes, collected from 18 phylogenetically distant fungi.** For each RNA-seq dataset (study), we calculated for each gene fold changes (FC) as the ratio between the expression in a condition of interest and the expression in the condition that was defined as the control in this study. Each line represents a condition of interest, each column a gene of interest (corresponding names of the genes are given in Figure 6). Genes have been distributed in three groups: the ‘R series’ that corresponds to 12 candidate reference genes pre-selected from *T. versatilis* data; the ‘C series’ that corresponds to more classic reference genes previously used in most of gene expression studies, including for filamentous fungi; and the ‘Sc series’ that corresponds to genes homologous to *S. cerevisiae* genes, which were previously validated as promising reference genes in this yeast species. Numbers reported in the heat map correspond to log _(base 2)_ of FC values. Colour scale and correspondences between Log _(base 2)_ and FC values are indicated in the legend (green colour set for a fold-change of 1 (log_2_ = 0); red colour arbitrary set for differential expression equal or higher than 12 (|log_2_| ≥ 3,5). Empty cells: data not available.

Changes in transcript levels in the different conditions for the different fungi, were used to generate a heat map (Figure 5). None of the genes from the ‘C series’ was stable in the two *T. versatilis* conditions and explains why they were not pre-selected in this study. Also, in RNA seq data collected from *R. irregularis*, *P. omphalodes*, *R. solani*, *F. radiculosa*, *M. oryzae* and *A. nidulans*, almost all the candidate genes and those from the ‘C series’ exhibited good expression stability, as indicated by the generalized greenish colour. Half of those genes had FC values lower than 1.2 (data not shown) and FC values almost never higher than 2. During the early stages of plant infection by *B. graminis*, extensive transcriptomic changes were observed for most of the genes. A similar situation was observed during different development stages of *S. macrospora,* where most of the reference genes were either down- or up-regulated. These two datasets strengthened the conclusion that even the best reference genes will never escape sporadic differential expression, so that validation of their stability is highly recommended prior to their use for normalization in each new project. When focused on the different *Aspergillus* and *Neurospora* species, visual inspection of the heat map suggested that genes from the ‘R series’ were slightly more stable. This was confirmed when pooling and analysing together all FC values from the ‘R’ and ‘C’ series, respectively, as the median and interquartile of these two gene subsets indicated a clear tendency to down regulation of the ‘C series’ (results not shown). A striking feature within this ‘R series’ was nevertheless the R5 (*Cu-ATPase*) gene that was strongly regulated under a few conditions, particularly in *N. crassa* exposed to Avicel or carbon starvation. This latter observation was however specific to *N. crassa*, as this gene was perfectly stable in *T. versatilis* mycelium similarly exposed to this carbon source or to C starvation (not shown). Finally, the fungal genes homologous to yeast Sc3 (*TFC1*) and Sc4 (*UBC6*) exhibited relatively stable transcript levels in most of the filamentous fungi and conditions of interest, with the notable exception of *B. graminis* during plant infection as already mentioned above. In contrast, Sc1 (*ALG9*) exhibited much higher fluctuations of FC values.

### Global analysis of reference genes stability in filamentous fungi

**Figure 6:**
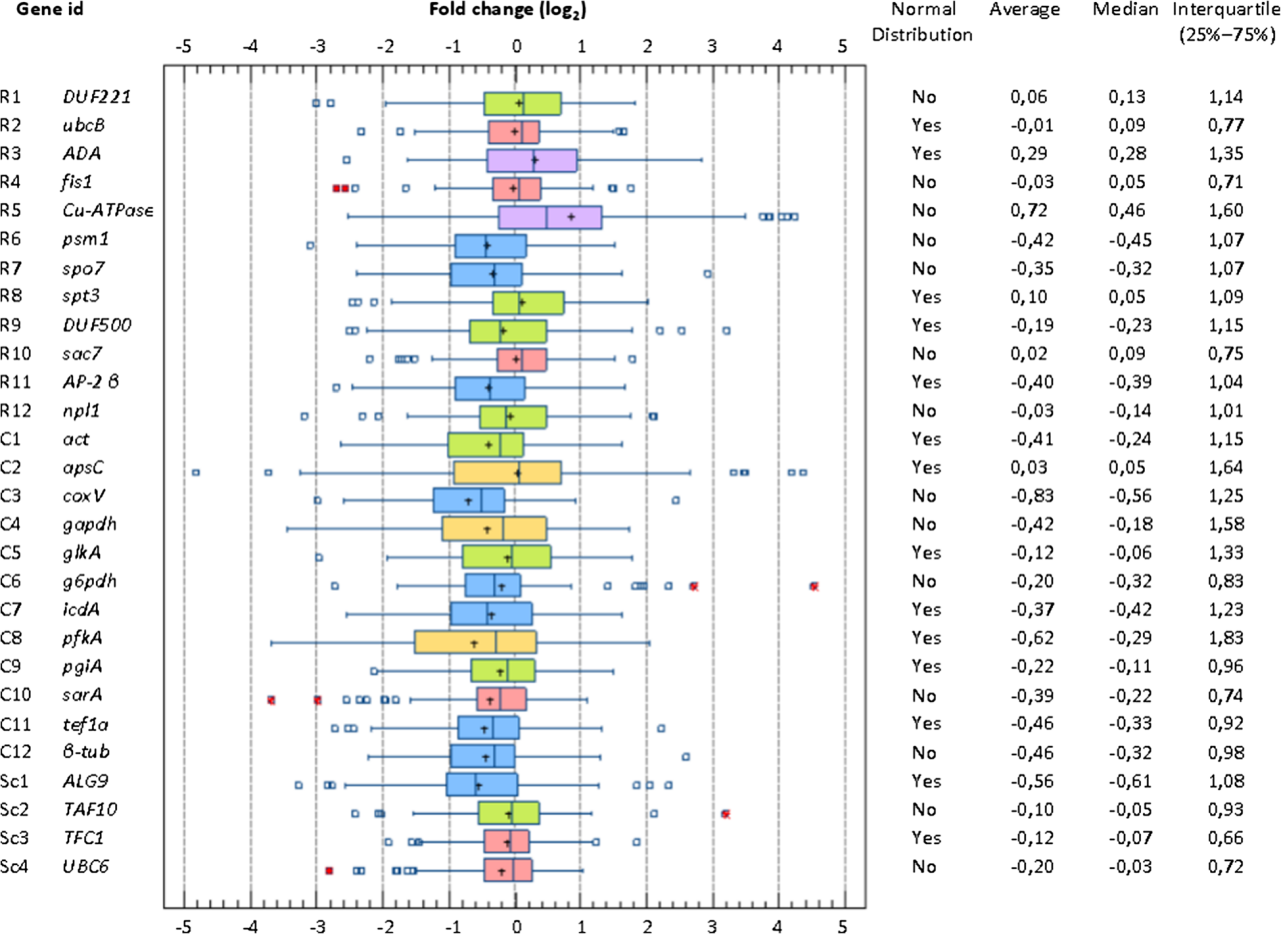
**Distribution of fold change values.** For each gene, the box-plot gathered about a hundred log_2_(FC) values presented in Figure 5 (Legend as in Figure 1). The extreme values amongst outliers are marked with a red asterisk. The colours of the boxes relate the classes that were determined from the HAC (see Figure 7). From the top to the bottom, we listed the new candidates (‘R series’), the classical reference genes (‘C series’) and the putative references homologous to *S. cerevisiae* genes (Sc1─Sc4). The right panel resumes the type of distribution (normal or not), average, median and interquartile for each gene.

To identify the most relevant reference genes among the whole ‘R’ and ‘C’ series, the 92 FC values were pooled for each gene independently (Figure 6). When looking at the median and interquartile, which are robust statistical parameters especially for small samples that are not normally distributed, the most promising genes should present a median close to zero and a compact interquartile, indicating no differential expression and low variation, respectively. Remarkably, R2 (*ubcB*), which was identified by geNorm as the best reference gene for *T. versatilis*, exhibited such requirements with a median close to zero and FC values that did not exceed 1.3 (repression or induction) in half of the conditions collected in this study. In contrast, the R5 gene (*Cu-ATPase*) exhibited a very strong bias towards overexpression and a much higher variation in FC values, even when removing outliers that mostly fitted with *N. crassa* samples exposed to cellulose.

**Figure 7:**
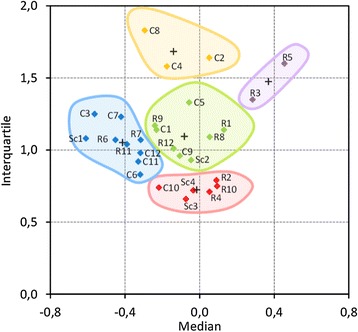
**Classification of the reference genes according to their median and interquartile.** Scatter plot of the interquartile *versus* median. The clusters that were obtained by hierarchical classification (HAC, see Additional file 7) are circled with different colours. The black crosses indicate the centroid of each cluster.

To search for groups of genes presenting similar behaviour, a cluster analysis (HAC) was conducted, using the median and the interquartile as variables (Additional file 7). Moderate partitioning led to the identification of 5 classes, highlighted in the interquartile *versus* median scatter plot (Figure 7). The best class (red), with its centroid having a median at zero and the lowest variation, contained three of the new candidates (R2 (*ubcB*) and R10 (*sac7*), previously designated as the best reference genes for *T. vesatilis*, and R4 (*fis1*)), C10 (*sarA*) that encodes a secretion-associated GTP-binding protein that was already identified as a good reference gene [35,36], and two genes homologous to *S. cerevisiae TFC1* (Sc3) and *UBC6* (Sc4). In contrast, the orange class, which had the largest interquartile of the study indicative of poor stability and higher probability of differential expression, included C8 (*pfk)*, C4 (*gapdh*) and C2 (*apsC*) genes. In between, the green category contained several classic reference genes such as C1 (*act*) and C5 (*glk*), which presented fairly centred medians but showed the highest variability within this class. Other frequently used reference genes such as C12 (*β-tub*), C11 (*tef1*) and C6 (*g6pdh*) were categorised in a less promising group that presented a reasonable level of variability but a tendency towards down-regulation (class 4, blue). Finally, R5 (*Cu-ATPase*) and R3 (*ADA*), which classified amongst the least stable genes in *T. versatilis* samples, confirmed their low stability in other filamentous fungi and a clear bias to overexpression (class 5, violet).

Even though genes in the red class could be considered as a very promising set of reference genes for normalization purposes in fungi, they too may present unexpected regulatory changes in specific contexts. An interesting case-study was indeed found in this work with two of these most promising reference genes, *i.e.* R4 (*fis1*) and Sc4 (*UBC6*), which were strongly down-regulated in *C. higginsianum* during infection phases of *Arabidopsis thaliana* (Figure 5). Moreover, although a reference gene is certified in an organism of interest, it does not preclude extremely different regulatory patterns in phylogenetically distant organisms exposed to strictly similar conditions. It was found for example that R5 (*Cu-ATPase*) was stable in *T. versatilis* exposed to Avicel or C starvation (data not shown), while it was strongly activated by these environmental conditions in *N. crassa*. Such examples clearly emphasise that validation experiments are mandatory to avoid the drawbacks of using inappropriate reference genes [77,78]. The second important point that should be stressed is the possible co-regulation of selected reference genes. While apparently linked to GTP, R10 (*sac7*) and C10 (*sarA*) seem to be implicated in independent functions, *i.e.* signal transduction and secretion, respectively. Unfortunately, this is not the case for R2 (*ubcB,* Ubiquitin-protein ligase activity) and Sc4 (*UBC6,* ER-associated protein catabolic process), which belong to a similar functional category and hence may show undesirable co-regulation. Therefore, the use of both of them as references genes should be discouraged, although both could be evaluated.

It is unlikely that expression of most of these promising reference genes will be found to be unstable simultaneously in future projects. If this was the case, it will require identifying and validating new genes. Transcriptomic data obtained from distant organisms studied under comparable conditions, or from the organism of interest cultivated in conditions as diverse as possible, could be collected. This strategy turned out to be successful in our hands for pre-selection of appropriate reference genes, even from a very limited set of transcriptomic data as starting material. The automated identification of suitable reference genes by the use of tools such as RefGenes [30] might be useful, taking care to focus on functionally unrelated candidates, provided that transcriptomic data that are targeted by this tool are generalised to all published datasets, including those that have been produced from filamentous fungi.

## Conclusions

Starting with a restricted set of 3 RNA-seq based transcriptomic datasets from *T. versatilis* cultivated under extremely different growth conditions, a list of 12 stable genes that belong to different functional classes was selected. The stability of transcript levels in more than thirty conditions of interest such as varied nutritional sources, stress exposure or time course analysis of conidial germination was examined. Three genes, R2 (*ubcB*), R10 (*sac7*) and R6 (*psm1*), were certified as the best reference genes for accurate normalization of expression levels in *T. versatilis*. Ideally, the optimal number of genes relies on the stepwise inclusion of additional reference genes until the time when this supplementary gene may not improve, nor worsen the normalization factor [33]. The overall good stability of the 12 candidates selected allowed us to show that any combination of 3 of them resulted in very similar normalized fold-change values and minimal normalization bias, even with the least stable genes on the list.

The main challenge concerning reference gene-based normalization is the circular problem in evaluating the expression stability of the candidate reference genes if no reliable normalization method is available [32]. To overcome this problem, transcriptomics can be used for pre-selection of unregulated candidates, choosing functionally unrelated genes to avoid co-regulated candidates, and identifying the best genes with the help of specific algorithms such as geNorm [33]. We clearly observed that normalization by single non-validated genes, *i.e. β-tub* or one of the least stable candidate genes of the list, introduced 3 to 8-fold normalization bias in more than half of the conditions investigated in this study. This could lead to inaccurate biological interpretation of gene regulation, particularly if the biological significance of subtle differences in fold-changes values of GOIs is to be considered.

Beyond their robustness in *T. versatilis*, the suitability of these reference genes for RT-qPCR analysis within the filamentous fungal kingdom was assessed, by collecting RNA-seq based transcriptomic data from 18 phylogenetically distant fungal species. The representative set of organisms and experimental conditions that was collected, confirmed that most of the classic “housekeeping” genes such as *g6pdh*, *β-tub* and *act* did not appear as the most stable genes, even if the latter has been classified among the best reference genes in few specific studies [35,41]. Other genes involved in central metabolism, *e.g. pfk*, *gapdh*, *aspC* or *glk*, also showed the highest occurrence of significant down- or up-regulation, which, together with their probable risk of co-regulation, strongly discouraged their further use as reference gene for RT-qPCR gene expression analysis.

The most promising group of six reference genes included *ubcB* (ubiquitin carrier protein), *sac7* (Rho GTPase activator), *fis1* (mitochondrial membrane fission protein), *sarA* (secretion associated GTP-binding protein), and two genes homologous to *S. cerevisiae TFC1* and *UBC6* (proteins involved in transcription initiation on Pol III promoters and ER-associated protein catabolic process, respectively). Four of these six genes – *sac7*, *fis1*, *sarA* and *UBC6 –* presented a non-normal distribution with rare cases of strong differential expression in these RNA-seq conditions. There is no single universal gene that exhibits stable expression levels in any sample and/or organism of interest [33]. The need for systematic validation of the stability of transcript levels from these reference genes in future studies is therefore warranted.

### Ethics

Material and experiments carried out in the frame of this article did not require any ethics approval.

## Additional files

Additional file 1:
**Table of culture conditions.**
Additional file 2:
**RNAseq RPKM values of studied reference genes.**
Additional file 3:
**Gene tags correspondence table.**
Additional file 4:
**RefFinder Output.**
Additional file 5:
**Detailed Heat Map.**
Additional file 6:
**List of classically used reference genes.**
Additional file 7:
**HAC tree.**
