# Supplementary material for: Tracking the best reference genes for RT-qPCR data normalization in filamentous fungi
Source: BMC Genomics. 2015 Feb 14;16(1):71. doi: 10.1186/s12864-015-1224-y (PMC4342825; doi:10.1186/s12864-015-1224-y)
Supplement: Additional file 1: — Table of culture conditions. [file 12864_2015_1224_MOESM1_ESM.docx]

Table of the different culture conditions to which *T. versatilis* was exposed.

| **RNA sample name ^a^** | **Corresponding number ^b^** | **Carbon source/ Stress condition** | **Concentration** | **Time of exposure** |
| --- | --- | --- | --- | --- |
| **AL1** | 0 | Glucose | 1% | 48 hours (inoculum) |
| **AL37** | 16 | Xylose | 0.2% | 30 minutes |
| **AL38** | 17 | Xylose | 0.2% | 2 hours |
| **AL39** | 18 | Xylose | 1% | 1 hour |
| **AL12** | 1 | Xylose | 1% | 24hours |
| **AL40** | 19 | Arabinose | 0.2% | 30 minutes |
| **AL41** | 20 | Arabinose | 0.2% | 2 hours |
| **AL42** | 21 | Arabinose | 1% | 1 hour |
| **AL14** | 2 | Arabinose | 1% | 24hours |
| **AL2** | 5 | Cellobiose | 0.2% | 30 minutes |
| **AL3** | 14 | Cellobiose | 0.2% | 2 hours |
| **AL5** | 22 | Thio-gentiobiose | 0.2% | 30 minutes |
| **AL6** | 23 | Thio-gentiobiose | 0.2% | 2 hours |
| **AL16** | 3 | Arbocel | 1% | 24 hours |
| **AL18** | 4 | Avicel | 1% | 24 hours |
| **AL20** | 6 | Xylan | 1% | 24 hours |
| **AL22** | 7 | Wheat straw | 1% | 24 hours |
| **AL24** | 8 | Wheat bran | 1% | 24 hours |
| **AL25** | 9 | 40°C |  | 1 hour |
| **AL26** | 10 | pH 2 |  | 1 hour |
| **AL27** | 11 | pH 8 |  | 1 hour |
| **AL28** | 12 | KCl | 0.5 M | 1 hour |
| **AL29** | 13 | N starvation |  | 1 hour |
| **AL30** | 15 | C starvation |  | 1 hour |
| **AL8** | 24 | Xylobiose | 0.2% | 30 minutes |
| **AL9** | 25 | Xylobiose | 0.2% | 2 hours |
| **AL10** | 26 | Xylobiose | 0.2% | 6 hours |
| **AL31** | 27 | Conidia on MM + glucose |  | 0 |
| **AL32** | 28 | Conidia on MM + glucose |  | 2 hours |
| **AL33** | 29 | Conidia on MM + glucose |  | 4 hours |
| **AL34** | 30 | Conidia on MM + glucose |  | 8 hours |
| **AL35** | 31 | Conidia on MM + glucose |  | 12 horus |
| **AL36** | 32 | Conidia on MM + glucose |  | 16 hours |

^a^ : Original RNA sample name

^b^ : Number given to each sample in Figure 4B
