# Supplementary figures and images for: Tracking the best reference genes for RT-qPCR data normalization in filamentous fungi
Source: BMC Genomics. 2015 Feb 14;16(1):71. doi: 10.1186/s12864-015-1224-y (PMC4342825; doi:10.1186/s12864-015-1224-y)

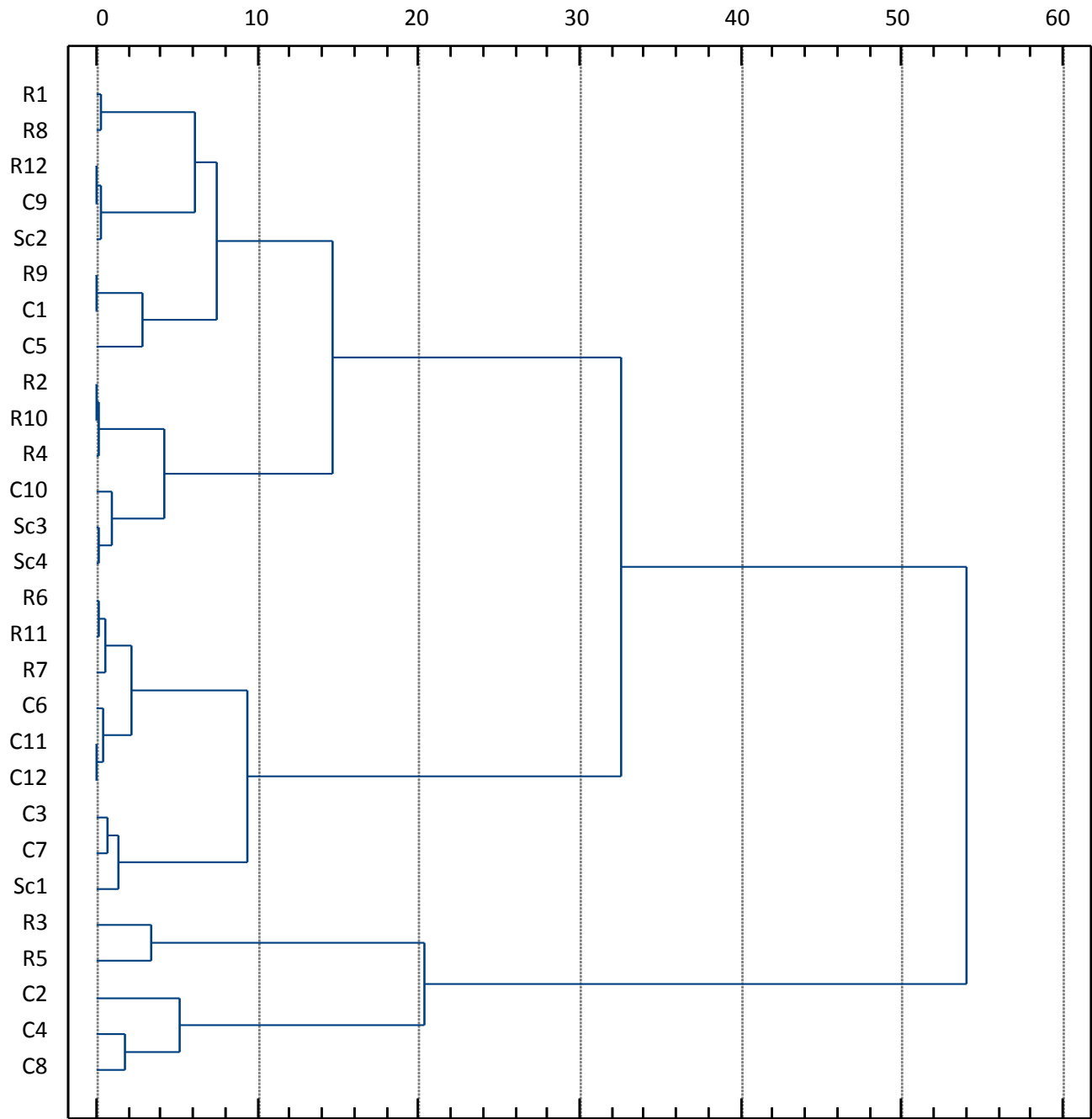

Supplement: Additional file 7: — HAC tree. [file 12864_2015_1224_MOESM7_ESM.pdf]
